# MACOP modular architecture with control primitives

**DOI:** 10.3389/fncom.2013.00099

**Published:** 2013-07-23

**Authors:** Tim Waegeman, Michiel Hermans, Benjamin Schrauwen

**Affiliations:** Department of Electronics and Information Systems, Ghent UniversityGhent, Belgium

**Keywords:** reservoir computing, echo state networks, motor primitives, movement primitives, motor control, MOSAIC, robot control

## Abstract

Walking, catching a ball and reaching are all tasks in which humans and animals exhibit advanced motor skills. Findings in biological research concerning motor control suggest a modular control hierarchy which combines movement/motor primitives into complex and natural movements. Engineers inspire their research on these findings in the quest for adaptive and skillful control for robots. In this work we propose a modular architecture with control primitives (MACOP) which uses a set of controllers, where each controller becomes specialized in a subregion of its joint and task-space. Instead of having a single controller being used in this subregion [such as MOSAIC (modular selection and identification for control) on which MACOP is inspired], MACOP relates more to the idea of continuously mixing a limited set of primitive controllers. By enforcing a set of desired properties on the mixing mechanism, a mixture of primitives emerges unsupervised which successfully solves the control task. We evaluate MACOP on a numerical model of a robot arm by training it to generate desired trajectories. We investigate how the tracking performance is affected by the number of controllers in MACOP and examine how the individual controllers and their generated control primitives contribute to solving the task. Furthermore, we show how MACOP compensates for the dynamic effects caused by a fixed control rate and the inertia of the robot.

## 1. Introduction

Catching a ball, reaching for a cup of coffee and drawing a figure on a blackboard are all tasks in which humans exhibit advanced motor control. We are able to perform such tasks robustly and adaptively, constantly anticipating an uncertain environment. Robots are most commonly used in environments which are fully deterministic, and are programmed in such a way that all possible situations are foreseen by the engineer. However, inspired by humans and biology in general, more and more techniques emerge in which robots can be used in dynamic environments without explicitly defining a set of rules to achieve advanced and adaptive motor control. The study of motor skills in nature also has sparked the interest of modular representations in both planned and actual motor commands. For instance, research performed on frogs (Bizzi et al., 1991) showed that electrical microstimulation of different areas of the lumbar cord generated distinct types of force fields in the frog's isometric leg movement. Similar research on frogs (Mussa-Ivaldi et al., 1994; Kargo and Giszter, 2000) and rats (Tresch and Bizzi, 1999) has shown that simultaneous stimulation of such areas result in a superposition of the separate recorded force fields, suggesting a modular control system. In Mussa-Ivaldi and Bizzi (2000), this work has been extended to the planning of limb movements and how to transform this planning into a sufficient set of motor commands.

Instead of using invasive and/or stimulation techniques to investigate the existence of a modular control system, researchers (d'Avella et al., 2003) also developed methods to find out if a large set of natural movements is the result of a combination of a limited set of *motor primitives*, solely based on muscle activity observations. By measuring such activations with Electromyography (EMG) and applying a decomposition technique over multiple EMG recordings, they found primitive representations, called synergies. These experiments were first conducted on frogs and later on humans (Hart and Giszter, 2004; Cheung et al., 2005; d'Avella and Bizzi, 2005).

Also on the behavioral level it has been demonstrated that humans try to follow mental templates of motion when executing a task (Bernstein, 1967). The presence of these mental templates or *movement primitives* can also be detected as velocity bumps (Doeringer and Hogan, 1998) during online movement corrections. A more detailed overview of primitives at the neural, dynamic and kinematic level can be found in Flash and Hochner (2005).

The idea of movement primitives also inspired research in robotics (Schaal et al., 2003). In Muelling et al. (2010) they demonstrate a robot that learned to play table tennis based on a set of primitives learned by imitating human table tennis movements. In Schaal et al. (2005), a flexible and reactive framework for motor control was presented which uses dynamic movement primitives (DMPs) (Schaal, 2006). This framework showed to be useful in the generation of walking motion of a biped based on oscillating DMPs or the generation of the swimming and walking motions of a salamander robot (Ijspeert et al., 2007) when DMPs are used as central pattern generator.

Most of these approaches define the primitives as oscillators or learned movements, and learn how to adapt them to get the desired objective. In this work, however, we take a different approach in which a similar decomposition emerges naturally, such that their combination converges to the objective. For this, we inspired our work on MOSAIC (modular selection and identification for control), originally proposed by Wolpert and Kawato (Wolpert and Kawato, 1998; Haruno et al., 2001) which suggest a feasible strategy on how a human motor control system learns and adapts to novel environments. For instance, when moving an empty cup or one filled with coffee. As both objects have different dynamics, MOSAIC learns a different controller for each object and assigns a “responsibility”-function, which allows for smooth switching between the controllers' individual contributions. When a new object is introduced, MOSAIC will generalize, by combining the contribution of each controller. To determine which controller should be used, each controller contains a forward model that predicts the next state of the object based on the previous control commands. If a controller's forward model is predicting well compared to the others, that controller is used. This architecture, however, can not be related to the idea of movement or motor primitives, as the number of controllers roughly depends on the number of objects, such that when handling a known object, a single controller's output is used.

Many variants of MOSAIC have been studied. The original implementation uses a gradient based method and later hidden Markov models. In Lonini et al. (2009), an alternative architecture based on locally weighted projection regression (LWPR) (Vijayakumar and Schaal, 2000) was presented which allows a better incremental learning of new tasks. Another approach (Oyama et al., 2001) uses a separate performance prediction network to determine which module should be used to learn the inverse kinematics of an arm. Likewise, in Nguyen-Tuong et al. (2009), they propose a localized version of Gaussian Process Regression in which a different model is trained for different regions in task-space.

The *modular architecture with control primitives* (MACOP) which we propose in this work is also inspired on MOSAIC. However, we want to build upon the idea of using a limited set of controllers whose contributions are continuously combined to produce the desired objective. Each controller's contribution should be mixed in a manner which permits all controllers to contribute to the objective, while still allowing for each controller to specialize in a part of the task. Due to the similarity with motion/motor primitives we will call the contributions of the individual controllers *control primitives*. We will provide a more detailed explanation on the similarities and differences with the common notion of primitives in the Discussion section of this paper.

Based on some interesting observations we omit the use of forward models (unlike MOSAIC) and use a simple heuristic to achieve the desired mixing mechanism which determines the “responsibility”. The controllers are constructed from Echo State Networks (ESNs) (Jaeger, 2001) which are inherently dynamic and therefore provide a natural platform for modeling and controlling a dynamic system such as a robot arm. After giving a description of MACOP we validate it by letting it learn the inverse kinematics (IK) of a 6 degrees-of-freedom (DOF) robot arm and we analyze its behavior. In the discussion we will address the following key points and differences with other techniques:

- Unsupervised learning of the mixing mechanism given some high-level requirements.
- Learning an inverse kinematic mapping which includes dynamical effects.
- How it allows to solve a task with control primitives where the complexity of a single controller is insufficient to solve the full task.
- How a control primitive compares with the notion of motor/motion primitives.

## 2. Materials and methods

### 2.1. Difficulty of controlling an unknown system

When controlling a system we want to find a way to control the states of that system by changing its input without actually knowing how the system internally works. All we can do is observe how the system responds to a certain input. Consider for instance the scenario in which we have a student trying to manipulate a cup of coffee with a robot arm. The actions that are under direct control of the student are the joint-torques, and this at a fixed control-rate. The variables that the student wishes to control, however, represents the trajectory of the cup of coffee. The student needs to use trial and error to learn to produce the desired result. In this process, he or she will need to implicitly learn the connection between his or her actions and the resulting trajectory of the cup. The main difficulties concerning this task can be summarized as:

- Learning an implicit mapping from actions to the resulting trajectory without prior knowledge.
- Handling redundancies because controlling multiple joints results in a mapping which is not unique.
- Because of a fixed control rate, the dynamic behavior of the robot needs to be anticipated.

Similar to this scenario, the system we use is a multi-jointed robot arm that needs to trace out a desired trajectory in its task-space.

### 2.2. Robot arm platform

In this work we perform all our experiments on a dynamic Webots simulation model of the PUMA 500 robot arm which has 6 DOF. This numerical model allows us to apply joint-angles and measure its actual values in the dynamical environment of the robot. We interact with this simulation model every 32 ms (default Webots configuration), which means that between every sample we take, 32 ms passed in the simulation environment, regardless of the computation time needed for the proposed algorithm. We control the joint-angles of the robot arm which are converted to joint-torques by PID (proportional-integral-derivative) controllers. Each joint is equipped with an encoder which allows us to measure the actual joint-angles. Additionally, the simulation environment provides us with the euclidean end-effector position of the robot arm.

### 2.3. MACOP

As mentioned before, controlling a system such as a robot arm poses some difficulties when the internal mechanisms of the robot are unknown. Although classical kinematic models are known for most commercial robots available, the increasing use of soft materials with passive compliant properties requires an adaptive modeling approach. Often a learning algorithm is used to create a model of the robot such that the model exhibits the same behavior (outcome) when perturbed by the same inputs (actions), which is called a forward model. By using for instance a neural network as model, the known structure of this network can be exploited to calculate a gradient which can be used to determine the actions that are needed to change the outcome as desired. Another approach is to learn an inverse model which maps a desired outcome to an action. However, learning such a model is difficult when the correct actions to a certain outcome are unknown. A more detailed overview on training and using such models can be found in Jordan and Rumelhart (1992).

Often the modeling complexity of a control problem can be reduced by decomposing the problem into less complex parts. For instance, if we again consider the cup lifting scenario for which we want to learn a model. This model will approximate the underlying dynamics of the particular task. If we extend this problem to lifting other objects, the resulting model needs to be able to approximate a single function that includes both tasks with different dynamics each.

One of the approaches that can solve such problems is called MOSAIC (Wolpert and Kawato, 1998; Haruno et al., 2001), which suggest a feasible strategy on how a human motor control system learns and adapts to new dynamic characteristics of the environment. MOSAIC learns a different controller for each different task, and uses a “responsibility”-function to decide which controller will be used, while still allowing for smooth switching between the controllers individual actions. When introducing a new task, MOSAIC will generalize, by combining the actions of each controller. To determine each controller's “responsibility”, every controller contains a forward model that predicts the next state of the object based on the previous control actions. If a controller's forward model is more accurate compared to the others, that controller's inverse model is trained further with the new observations and used to control the robot arm.

One potential weakness of this approach is that the performance of a forward model is not necessarily a good indicator of the modeling performance of the inverse model. To confirm this we have tried an approach directly based on MOSAIC. For each controller, we trained a reservoir (see section 2.4.2) to serve as both an inverse and forward model at the same time. We found that all forward models had initially roughly the same prediction error, leading to an equal responsibility factor for each controller as a result. During the training phase, however, small variations in performance error influenced both the training of the forward and the inverse models. Eventually this always causes one controller to be fully responsible at all times, making the other controllers redundant. These findings confirm the observations made in Haruno et al. (2001) even though in the original MOSAIC setup, the inverse and forward models are completely separate from each other, meaning that there is no relation at all between the modeling performance of the inverse and forward model. Based on these experiments one can argue that the responsibility of a controller is fully determined by noise on the forward modeling performance of a controller. Any other controller selection mechanism might thus be as useful as the one used by MOSAIC.

**Figure 1:**
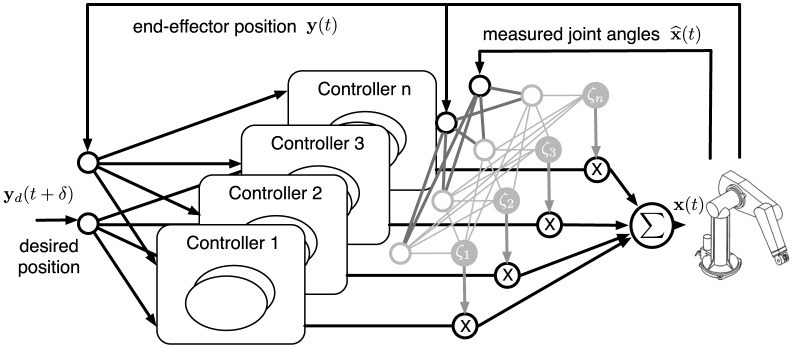
**Illustration of MACOP which consists of an ensemble of controllers depicted in Figure 2.** The desired (objective) and the current end-effector position are used as external inputs to each controller. The controller outputs are weighted by a scaling factor ζ_*i*_ and superimposed with each other such that the resulting joint-angles control the robot. The used ζ_*i*_ represent the responsibility of a controller and is determined by the measured joint-angles and end-effector position.

Therefore we propose a Modular Architecture for Control with Primitives (MACOP) which is inspired on MOSAIC, and depicted in Figure 1. Instead of using both an inverse and forward model, we only use inverse models to produce actions for our robot arm. Determining when a controller (inverse model) should contribute to the task is learned unsupervised given the robot's state and some desired mixing properties. This can be related to a Kohonen map (Kohonen, 1998).

#### 2.3.1. Controller selection

As depicted in Figure 1, the actual control signal is a weighted sum of the outputs of a limited number of controller outputs. The weight (or scaling) factors (which are the equivalent of MOSAIC's “responsibility”), depend on observable properties of the robot. Each controller learns to control the robot arm by creating an inverse robot model. Simultaneously, the mixing mechanism is trained, also online. In order for a controller to distinguish itself from others, the rate at which each controller is trained will be modulated according to its corresponding responsibility. This will be explained in more detail when we describe the operation of a single controller.

Suppose we have *N*_*c*_ controllers. We denote the output of the *i*-th controller as **x**_*i*_(*t*). The controlled joint-angles **x**(*t*) are then given by:

(1) $$x(t)=\sum_{i=1}^{N_{c}}\zeta_{i}(t)x_{i}(t),$$

where ζ_*i*_(*t*) is the scaling factor, or “responsibility” of a controller, which decides how much each controller is expressed in the final control signal. Ideally, we would like to let ζ_*i*_(*t*) express the momentary accuracy of each controller. For example, if each controller is randomly initialized before training, certain controllers may be better than others when the arm is near a certain pose, and we would like to use the ζ_*i*_ to scale up the control signal of these controllers, and suppress that of the others. In reality, however, we cannot directly measure the accuracy of each individual controller, as the robot is driven with the weighted sum of the control signals, and not the individual controllers.

Therefore, we will apply a different strategy. We will introduce a way in which the scaling factors will automatically start to represent local parts of the operating regime of the robot, and next we will specialize the associated controllers to be more accurate within this local area.

We wish for ζ_*i*_(*t*) to only depend on the current end-effector position **y**(*t*) and the measured joint-angles $\hat{x}(t)$, both of which are observable properties of the robot arm. As each controller will attempt to learn an inverse model, the combined control signal will need to be of the same magnitude as the individual control signals. Therefore, we will make sure that the scaling factors are always positive, and always sum to one:

(2) $$\sum_{i=1}^{N_{c}}\zeta_{i}(t)=1\text{ and }0\leq \zeta_{i}(t)\leq 1.$$

Both these qualities can be ensured if ζ_*i*_ is calculated by a *softmax* function. First we use a linear projection from the joint-angles and end-effector position to a vector **r**:

(3) $$r(t)=\left[\begin{array}{c} r_{1}(t)\\ r_{2}(t)\\ \vdots \\ r_{N_{c}}(t) \end{array}\right]=V(t)\left[\begin{array}{c} y(t)\\ \hat{x}(t) \end{array}\right],$$

Next, we compute the softmax function.

(4) $$\zeta_{i}(t)=\frac{\exp(r_{i}(t))}{\sum_{j=1}^{N_{c}}\exp(r_{j}(t))},$$

The projection matrix **V**(*t*) is a matrix of size *N*_*c*_ by $N_{y}+N_{\hat{x}}$, where *N*_**y**_ and $N_{\hat{x}}$ are the dimensions of **y**(*t*) and $\hat{x}(t)$, respectively. Dependence on time comes from the fact that, like all parameters, **V** is trained online.

**V**(*t*) is randomly initialized, with elements drawn from a normal distribution 
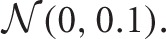
 It will determine how the responsibilities are distributed. We need to train **V**(*t*) in such a way that MACOP learns to generate the target trajectories by mixing all contributions as we desire. In this work we wish to obtain the following two qualitative properties:

- Each controller should contribute in a unique way to the movement generation of the robot. In order for a controller to distinguish itself from the others, its corresponding ζ_*i*_(*t*) should peak over the others. We wish that there is sufficient variation, such that at each moment in time some controllers are significantly more responsible than others.
- On the other hand we wish to make sure that there are no responsibilities that are close to zero at all times, such that all controllers are put to good use, and we fully exploit the potential power of the ensemble. We wish to avoid the situation we observed when we implemented the MOSAIC-based controller ensemble, where eventually only one controller contributed to the task.

Based on these two desired properties, we will construct a learning algorithm for training **V**(*t*). The first property of our mixing mechanism can be achieved by gradually increasing the magnitude of **V**. This results in a more strongly peaked distribution for the scaling factors. This can be understood by looking at the limit situations. If all elements of **V**(*t*) are zero, all scaling factors are equal. If the magnitude goes to infinity, the softmax function will be equal to one for the highest element, and zero for all others. Controlling the magnitude of **V**(*t*) allows us to make a smooth transition between these extremes. We chose to increase the magnitude of **V**(*t*) linearly each time step by adding a small increment, equal to **V**(*t*) divided by its Frobenius norm.

The second mixing property requires that all controllers contributes significantly to the robot motion. The manner in which we chose to do this was to suppress the scaling of the momentary maximal scaling factor. This ensures that no single scaling factor can remain dominant for a long time. Suppressing one scaling factor automatically scales up the others, such that in the end none of the scaling factors remains very small at all times. In order to train **V**(*t*) to get this effect, we need to set target values for the ζ_*i*_(*t*) at each time step. We set the target value of the highest ζ_*i*_(*t*) equal to *N*^−1^_*c*_ (which would be the long-term time average of all scaling factors if they all contribute equally). At the same time we have to make sure that the sum of the target values is equal to one (i.e., a target that can be reached by a softmax function). To obtain this, the target values of the other ζ_*i*_(*t*) are equal to themselves, scaled up to ensure that the sum of the targets equals one. If we denote the target value for ζ_*i*_(*t*) as θ_*i*_(*t*), we can write

(5) $$\theta_{i}(t)=\{\begin{array}{ll} h(t)\zeta_{i}(t), & \text{if }i\neq \text{argmax}(\zeta_{i}(t))\\ \frac{1}{N_{c}} & \text{if }i=\text{argmax}(\zeta_{i}(t)) \end{array},$$

with

(6) $$h(t)=\frac{1-N_{c}^{-1}}{1-\max(\zeta_{i}(t))}.$$

To train **V** according to these target values, we calculate the gradient of the cross-entropy^1^ *H*(θ_*i*_, ζ_*i*_) with respect to **V**. For both desired properties we have defined an update rule and each time step we add up both contributions, resulting in the following update rule:

(7) $$V(t+1)=V(t)+\eta_{g}\frac{V(t)}{||V(t)|{|}_{F}}+\eta_{s}[y^{⊤}(t),{\hat{x}}^{⊤}(t)](\zeta (t)-\theta (t)),$$

where ζ(*t*) and θ(*t*) are column vectors with the responsibility factors and their targets, respectively, and η_*g*_ and η_*s*_ are two learning rates. In order to prevent one mixing property to dominate the other, we set these learning rates such that both properties are present. Left on its own, Equation (7) never converges. What will happen is that the magnitude of **V** slowly keeps on increasing, and in the long term, the scaling factor distribution will become highly peaked (at each moment, one will be close to one, the others close to zero). Therefore, during all our experiments, unless mentioned differently, we calculate the root mean-square-error (RMSE) between the desired and the measured end-effector position^2^ over a moving time-window of 1000 samples. When this RMSE becomes smaller than 1 mm we start to linearly decrease both η_*g*_ and η_*s*_ over the course of 5000 samples until they reach 0. After this point the elements of **V** no longer change.

### 2.4. Single controller

As described before, we will use inverse models for controlling our multi-jointed robot arm. Rather than finding the mapping from joint-angles (actions) to end-effector position (outcome), we will approximate the inverse mapping: from outcome to action. Essentially, this means that we train a model to directly provide us with the correct joint-angles for any given desired end-effector position.

#### 2.4.1. General setup

**Figure 2:**
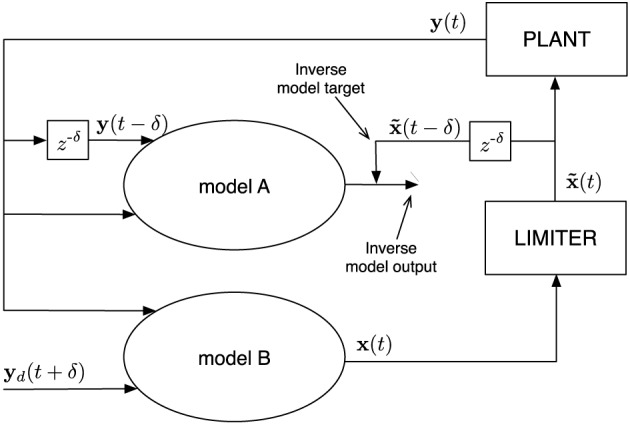
**Schematic representation of a single controller.** Model A and B are identical at every moment in time, but receive different input signals. The optional limiter limits the values **x**(*t*) to a desired range which, for example, represent imposed motor characteristics. Afterwards, the limited values $\tilde{x}(t)$ excite the plant (robot in this work). The signal $\tilde{x}(t-\delta )$ is the desired output which model A is trained to generate from the plant output, i.e., it learns the inverse model. This inverse model is then simultaneously employed as a controller to drive the plant (model B), which receives a desired future plant state **y**_*d*_(*t* + δ) as input, instead of the actual one.

As described before, the end-effector position (outcome) is denoted by **y**(*t*), and the joint-angles (the actions) by **x**(*t*). We assume that we can train a model (model A in Figure 2) to approximate the past joint-angles **x**(*t* − δ), δ being a fixed delay period, given that it receives the current and the delayed end-effector position **y**(*t*) and **y**(*t* − δ), respectively. This part of the control mechanism is the inverse model.

Simultaneous with training the inverse model, we use it as a controller. In order to do this, we use an identical copy of the model (model B in Figure 2), which has as input the current end-effector position **y**(*t*), and a desired future end-effector position **y**_*d*_(*t* + δ). This model, given that the inverse model performs sufficiently well, provides the required joint-angles **x**(*t*) to reach the set target after the delay. For some plants it might be necessary to limit these values to a certain range. For instance, when controlling a joint, the angle in which it can be positioned is bounded. In Figure 2 this bounding is represented by a limiter which converts **x**(*t*) values to $\tilde{x}(t)$.

In general, the optimal δ depends on the rate at which the dynamics are observed (sample rate) and the kind of dynamics (fast or slow) that are inherent to the control task. Plants (the system under control) with fast dynamics usually require a smaller δ than slower dynamical systems when using the same sample rate. In this work we chose δ = 1, because one time step delay is sufficient to capture the dynamics of the task at hand. More details concerning this parameter can be found in Waegeman et al. (2012).

In order to train the inverse model we will use online learning, i.e., the inverse model is trained during operation. Initially the untrained inverse model will not be able to provide the desired joint-angles, and the driving signal **x**(*t*) is essentially random. Model A will use the resulting end-effector position to learn what signal was provided by model B. The random driving signal will assure that in this initial training phase model A is provided with a sufficiently broad set of examples. However, to further improve exploration and to speed up training in the initial training phase, we add a small amount of noise [initially drawn from 
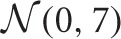
 in mm] to the desired end-effector position, of which the standard deviation linearly diminishes to 0 over the course of 50,000 samples of training. As soon as model A becomes sufficiently accurate, model B will begin providing the actions for obtaining the desired end-effector positions. In this phase, the inverse model will learn to become especially accurate in the operating regime in which the robot provides the resulting end-effector positions.

In principle, there is no need to ever stop training the inverse model in the proposed controller. Indeed, if the experimenter knows that the conditions of the setup may change over time, it could be desirable to keep the online learning mechanism active at all times in order to let it keep track of changes in the system. For this paper, however, we chose to gradually slow down the learning algorithm and at some point in time let it stop, such that all parameters in the controller architecture remain fixed during testing. More details are provided in section 2.4.4.

#### 2.4.2. Echo state networks

**Figure 3:**
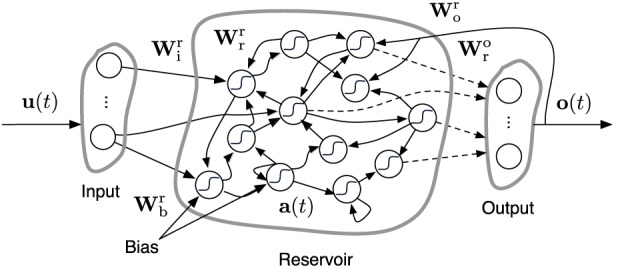
**Description of an ESN.** Dashed arrows are the connections which can be trained. Solid arrows are fixed. **W**^*k*^_*g*_ is a matrix representing the connections from *g* to *k*, which stand for any of the letters *r, i, o, b* denoting *reservoir, input, output*, and *bias*, respectively. **u**(*t*), **o**(*t*) and **a**(*t*) represent the input, output, and reservoir states, respectively.

We use an *Echo State Network* (ESN) (Jaeger, 2001) as inverse model. An ESN is composed of a discrete-time recurrent neural network [commonly called the *reservoir* because ESNs belong to the class of Reservoir Computing techniques (Schrauwen et al., 2007)] and a linear readout layer which maps the state of the reservoir to the desired output. A schematic overview of this is given in Figure 3. An ESN has an internal state **a**(*t*) (often referred to as reservoir state), which evolves as follows:

(8) $$a(t+1)=\tanh\left(W_{\text{r}}^{\text{r}}a(t)+W_{\text{i}}^{\text{r}}u(t)+W_{\text{o}}^{\text{r}}o(t)+W_{\text{b}}^{\text{r}}\right).$$

Here, **u**(*t*) is the reservoir input signal and **o**(*t*) the output produced by the ESN (see below). The weight matrices **W**^*k*^_*g*_ represent the connections from *g* to *k* between the nodes of the network, where *r, i, o, b* denote *reservoir, input, output*, and *bias*, respectively. In the case of the controller setup we discussed earlier, the reservoir input **u**(*t*) of model A consists of the concatenation of the past and present end-effector positions, and that of model B of the present and desired end-effector position. During our experiments we scale the input and training signal to the ESN such that their values are between −1 and 1. Consequently, we will need to undo this scaling before the generated network output represents actual joint-angles which can control the robot.

The ESN output **o**(*t*) is generated by:

(9) $$o(t)=W_{\text{r}}^{\text{o}}(t)a(t),$$

i.e., a linear transformation of the reservoir state over **W**^o^_r_(*t*). The core idea of ESNs is that only this weight matrix is explicitly trained. The other weight matrices have fixed, randomly chosen elements of which only the global scaling is set. As we use a continuously operating online learning strategy, **W**^o^_r_(*t*) is time depending. In the more common ESN setup they are trained offline using, e.g., ridge regression, and remain fixed once they are determined.

Each ESN in this paper is initialized by choosing the elements of **W**^r^_r_, **W**^r^_i_, and **W**^r^_b_ from a standard normal distribution 
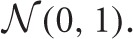
 Next, **W**^r^_r_ is scaled such that its spectral radius equals one, and the matrices **W**^r^_o_, **W**^r^_i_ and **W**^r^_b_ are multiplied with a factor 0.1 (hand tuned).

The reservoir serves as a random non-linear dynamical system which can extract useful information of its input. Due to its recursion, a reservoir has *fading memory*, i.e., it retains information of the past input signal but gradually forgets it over time and allows ESNs to be used for processing time series.

#### 2.4.3. Linear controllers

In order to check how much the operation of MACOP depends on the type of controller, we also conducted an experiments in which we used linear controllers. Here, the output of the inverse model is a direct linear combination of its input, so no non-linearity or memory is present in these controllers, and learning the non-linear part of the full inverse kinematics will largely need to be accounted for by training the scaling factors. Here too, we will train the system online, according to the algorithm described in section 2.4.4.

#### 2.4.4. Recursive least squares

In order to train the inverse model online we will use Recursive Least Squares. With each iteration the output weights are adjusted such that the network converges to the desired output. However, the rate at which these weights are changed is controlled by the corresponding responsibility factor ζ_*i*_. Within the proposed MACOP architecture, such adaptive learning rate allows each controller's inverse model to distinguish itself from the other controllers. Additionally, in order to allow the weights to converge to fixed values, the training speed is modulated with a factor *l*(*t*). Because our description of a controller is equal for all controllers within MACOP we will omit the use of the index *i* which refers to the *i*-th controller. We can therefore write the weight update equation as follows:

(10) $$W_{r}^{o}(t)=W_{r}^{o}(t-1)-l(t)\zeta (t)e(t){\left(Q(t)a(t)\right)}^{⊤},$$

where

(11) $$Q(t)=\frac{Q(t-1)}{\lambda }-\frac{Q(t-1)a(t)a^{T}(t)Q(t-1)}{\lambda (\lambda +a^{T}(t)Q(t-1)a(t))},$$

and

(12) $$Q(0)=\frac{I}{\alpha }.$$

Here, **a**(*t*) are the current states, λ a forgetting factor and α an initially chosen value. **Q**(*t*) is a running estimate of the Moore–Penrose pseudo inverse (**a**^*T*^**a**)^−1^, (Penrose, 2008). **Q**(0) denotes the initial value of **Q**. The error **e**(*t*) is the difference between the actual and the desired joint-angles. To allow **W**^**o**^_**r**_(*t*) to converge together with the projection matrix **V** from Equation (7), *l*(*t*) is decreased linearly from 1 to 0 in the same fashion as the learning rates η_*g*_ and η_*s*_ in Equation (7), i.e., as long as the average error over the last 1000 time steps is larger than 1 mm, it is equal to one, and as soon as it is smaller, it linearly decreases to zero over the course of 5000 time steps.

### 2.5. Analyzing MACOP

Each controller learns to produce a set of joint-angles online, which contribute to solving the IK problem. We define such a set of joint-angles as being a control primitive. Mixing these primitives results in a set of joint-angles to which the robot is positioned. To analyze each controller and its contribution we define several methods which we will describe in the remainder of this section. The results of these analyses can be found in section 3.

#### 2.5.1. Tracking a trajectory

As described above we designed MACOP such that the scaling of a controller depends on the location of the end-effector and the robot's pose. A control primitive that has the largest “responsibility” [biggest ζ_*i*_(*t*)], we will call the dominant primitive (generated by the dominant controller). Furthermore, we study the time course of the scaling factors, and how they relate to the motion of the robot. We color the trajectory of the end-effector according to which controller is the dominant one at that position, this in order to show which controllers specialize in which regions of task-space. We show the resulting trajectories using both ESN and linear controllers.

#### 2.5.2. Selecting a single controller: control primitives

Even when the scaling factors strongly fluctuate in time, this does not necessarily mean that the controllers are sending different control signals. Indeed, even though the learning speed is modulated according to the scaling factors, all of them still are trained to perform the same task. In order to verify if specialization indeed occurs, we conduct experiments in which after the training phase, only one controller is used (i.e., we set its scaling factor to 1 and all others to 0). We show the resulting trajectories, and we study individual model performance compared to its true scaling factor.

#### 2.5.3. One vs. multiple controllers

One of the main research questions of this paper is of course how much we can profit from using MACOP versus a single controller. In order to answer this, we measure how well the setup performs as a function of the number of controllers. In order to keep this comparison fair, we make sure that for each setup the number of trainable parameters (the total number of output weights of the ESN) remains constant.

### 2.6. Dynamic effects of kinematic control

**Figure 4:**
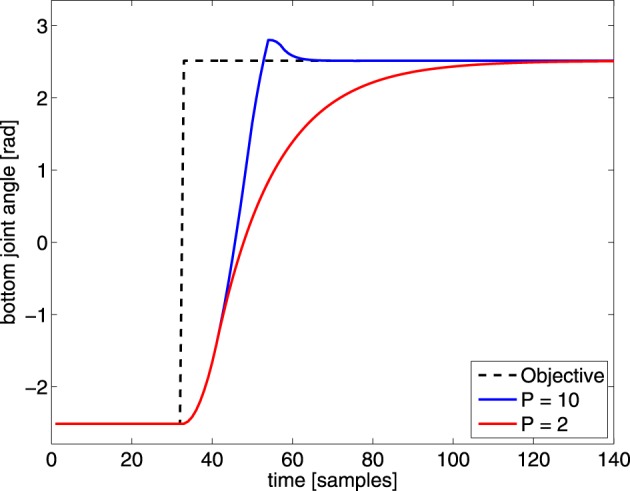
**Illustration of a sudden change in desired joint-angle of the bottom joint (black dashed line) of the PUMA 500.** The robot response is shown for this particular joint with different *P*-parameters, to illustrate the effect of a changed *P* value. By decreasing the *P*-parameter the dynamic transition time from one position to the other increases.

An inverse kinematic mapping maps a desired task-space position to the corresponding joint-angles. In most evaluations of learning inverse kinematics, a new command is only sent when the previous desired joint-angles are reached. For this, a direct inverse mapping without memory suffices. However, in our experiments we do not wait for the robot to reach the commanded joint-angles and send new joint-angles at a constant rate (every 32 ms). As shown in Figure 4, a PID-controller, which applies the necessary torque to reach a desired joint-angle, has a dynamic transition before reaching a new target angle. These dynamic transitions need to be compensated by the controllers as well, in order to reach the desired outcome in time^3^. Such transitions require memory instead of a direct mapping. We evaluate the MACOP's ability to cope with these dynamic effects by changing the *P*-parameter of each joint's PID-controller (which determines how fast the robot can react to changes in the desired joint-angles) such that these dynamic effects become more important.

## 3. Results

All training parameters for the experiment are provided in Table 1. The RLS parameters λ and α we chose based on previous experience. The learning rates η_*g*_ and η_*s*_ we found by trial and error, but we experimentally verified that performance does not change much in a broad region around the provided values.

**Table 1:** **Simulation parameters**.

**Parameter**	**Value**
λ	1 − 10^−4^
α	0.01
η_*g*_	0.00008
η_*s*_	0.0002

### 3.1. Tracking a trajectory

**Figure 5:**
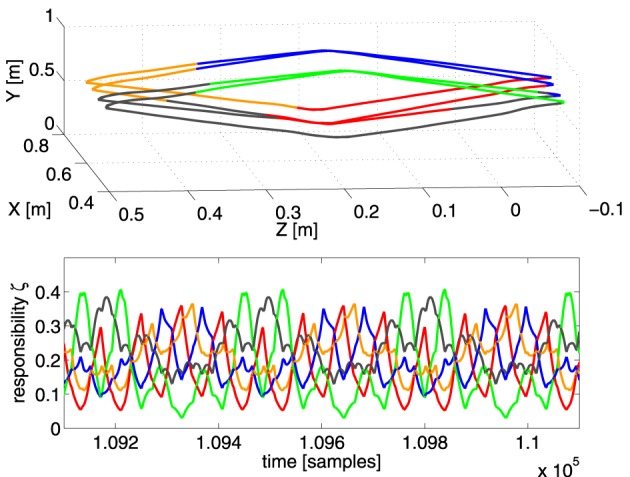
**Top panel:** the resulting end-effector trajectory generated by the robot arm for the rectangular target trajectory after convergence (RMSE < 1 mm for the full trajectory). The corresponding color of the dominant controller is shown. **Bottom panel:** the responsibility factors ζ_*i*_(*t*) as a function of time.

**Figure 6:**
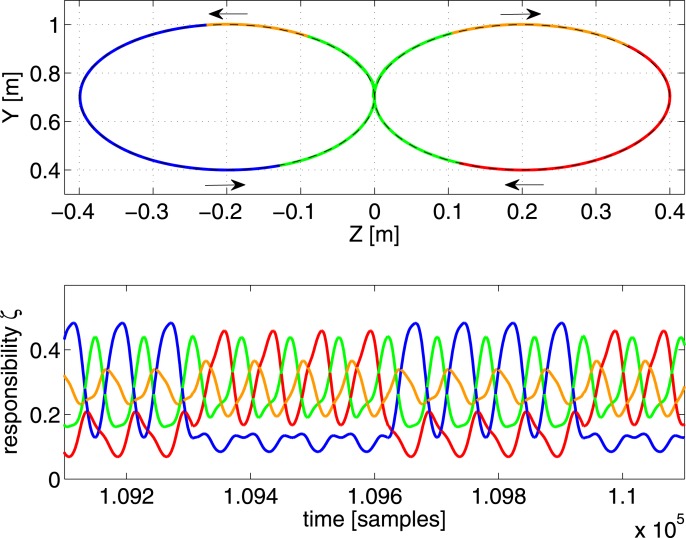
**Top panel:** the resulting end-effector trajectory generated by the robot arm for the circular target trajectory after convergence. The corresponding color of the dominant controller is shown. The arrows indicate the direction of the trajectory. **Bottom panel:** the responsibility factors ζ_*i*_(*t*) as a function of time.

To investigate the overall behavior of MACOP, we applied multiple desired trajectories of the robot end-effector. In both Figures 5, 6, we show the resulting trajectories (after convergence) of following a rectangular and circular-shaped target trajectory. In both experiments an RMSE of 10 cm was achieved within 10,000 samples and a RMSE of 1 mm (point of convergence) within 100,000 samples, demonstrating that the system is able to follow a desired trajectory closely.

In the first experiment, we train the robot to generate a rectangular trajectory which spirals back and forth into the X-direction over several passes. For this we used 5 controllers, each with 50 neurons. In the top panel of Figure 5 we show the trajectory generated by the robot after convergence (all learning rates are 0 and RMSE = 1 mm). Each part of the trajectory is colored according to which controller is dominant (has the maximum scaling factor) at that time. The responsibilities ζ_*i*_(*t*) themselves are shown in the bottom panel of Figure 5. It appears that the 5 controllers have formed a specialization for certain regions of task-space, their responsibilities ζ_*i*_ peaking at the corresponding parts of the trajectory.

Notice that the depth of the trajectory in the X-direction is rather small (20 cm), and yet the scaling factors strongly vary as a function of it (as is especially apparent in the green and blue parts of the trajectory). This strong change in scaling factor is caused mostly by the pose of the robot, and not so much the end-effector position, as we have verified by experimentally testing the sensitivity of the scaling factors as a function of the joint-angles and position. This suggest that the control architecture effectively uses information of the robot pose to solve the task.

A second experiment (*N*_*c*_ = 4, 50 neurons each) extends the difficulty of the previous trajectory to demonstrate responsibility/task-space correlations over a larger time period of the desired movement. The trajectory describes four passes of a circle in a single direction during 8 s, after which the trajectory smoothly switches to describing a shifted circle in the opposite direction of the previous circle.

**Figure 7:**
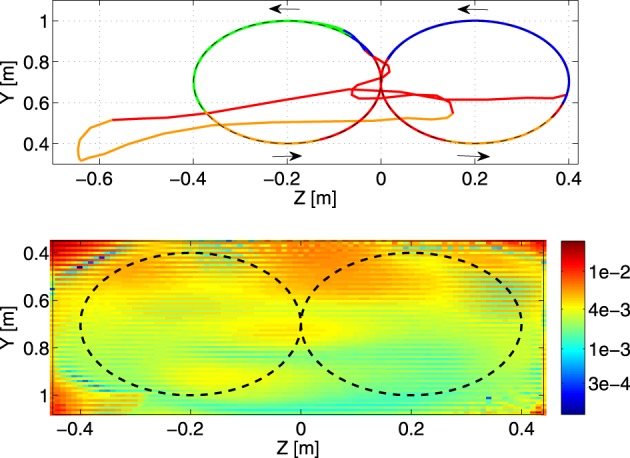
**Top panel:** a part of the generated trajectory before the switch and after a jump, where the dominant controllers are represented by a corresponding color. The direction in which the trajectory is tracked is indicated by the arrows. **Bottom panel:** visualization of the generalization performance of the learned IK (circular training trajectory: dashed line) on a test grid. Color scale is the RMSE in m.

We show the result after convergence in Figure 6. The rotation direction in which the trajectory is followed is indicated by the arrows. In the left part of the circular trajectory the blue controller is contributing a significant part to the control of the robot. This contribution is reduced when the robot is performing the right circular movement. In this part of the trajectory, the red controller is contributing more. In order to verify MACOPs robustness we consider the double circle trajectory after training, and suddenly jump ahead in time in the desired trajectory such that there is a discontinuous jump in the target end-effector position. The result is shown in Figure 7. It appears that after a large initial overshoot, the robot recovers and is eventually capable of tracking the desired trajectory again. The overshoot can be largely explained by the fact that the controller never saw a discontinuous jump during training, such that it has seen no examples of what happens when large torques are applied on the joints. Furthermore, the sudden jump forces the robot arm into a region of task-space where it never resided during training, causing unpredictable behavior.

After training MACOP (same configuration as before) with the double circle trajectory we define a test grid on the plane of the training trajectory with a resolution of 1 cm. The test target points of this trajectory are visited by sweeping the grid back and forth in the Z-direction. The result of such an experiment is shown in the bottom panel of Figure 7. Each pixel represents the RMSE (in meters) of a specific grid point. Averaged over 10 experiments (different initialization and training) a mean RMSE = 4.4 mm with a standard deviation of 3.1 mm is achieved. Note that the RMSE in the grid corners are bigger because they are harder to reach.

**Figure 8:**
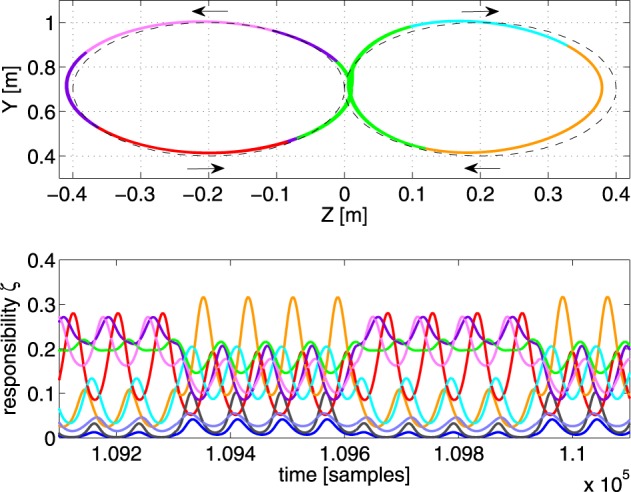
**Illustration of the tracking performance of MACOP with 9 linear controllers after convergence. Top panel:** the resulting trajectory, colored according to the dominant controller. The dashed line is the target. **Bottom panel:** the corresponding responsibilities as a function of time.

In the final experiment of this paragraph, we tried a set of linear controllers to see if MACOP is able to still control the robot arm to generate a trajectory with very low-complexity controllers. We found that we need at least 9 controllers to approximate the target trajectory with a final RMSE = 1.5 cm. Using MACOP with fewer controllers does not work. Figure 8 shows the resulting trajectory and the scaling factors of the individual controllers. The fact that MACOP is capable of solving the tracking task with such basic controllers is a strong indicator that the presented training algorithm for the scaling factors is quite successful in distributing the complexity of the full task. It also demonstrates that MACOP can be easily extended to include any kind of inverse model.

### 3.2. One vs. multiple controllers

One of the main assumptions underlying MACOP is that we assume it is beneficial to distribute the full control task over multiple controllers. The first check we performed was to make sure that the mixing using the softmax function is in fact responsible for the increase in performance, and not just having several distinct controllers in the first place. We have tested this by keeping the responsibility factor ζ_*i*_(*t*) constant and equal to *N*^−1^_*c*_ for each model, and this in the case of 5 ESN-controllers. It turns out that this situation leads to the same performance that is attained by using a single, large reservoir (which performs worse, as we will show next), showing that the variable responsibility factors directly increases performance.

To investigate how much the tracking performance depends on the number of controllers we conducted an experiment in which we measure the mean error on the trajectory for different number of controllers. We measure the distance from the end-effector to the target averaged over 5000 samples after training. We linearly reduce all learning rates to 0 after 100,000 samples of training (because some experiments will never reach the requirement of an RMSE less than 1 mm). For an increasing number of controllers we apply MACOP on trajectories which are based on all 26 letters in the English alphabet, which we have drawn by hand and of which we recorded position as a function of time. As the trajectory is repeated periodically, we also made sure that the end and starting point are the same in each trajectory (to avoid sudden jumps). After recording we scaled the trajectories and placed them in the YZ-plane in reach of the robot.

**Figure 9:**
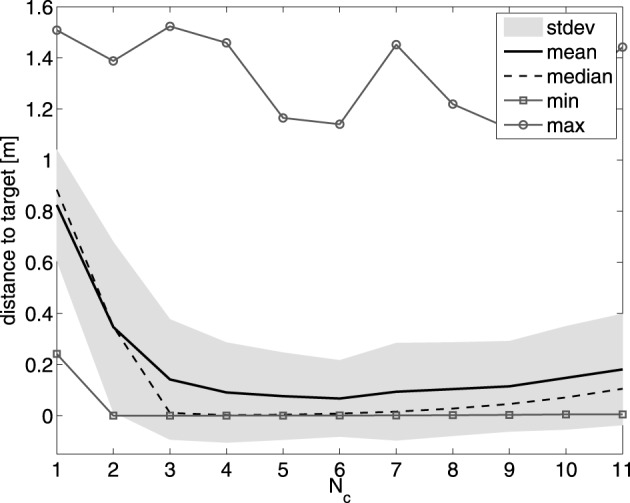
**Effect of the number of controllers on the tracking performance of MACOP on the English alphabet trajectories.** The mean, median, standard deviation, minimum and maximum values over 1300 experiments are shown for each controller configuration.

In each experiment we train a randomly initialized controller ensemble to produce a single letter, and we measure the RMSE after convergence. For each number of controllers, we measure the average RMSE over 50 instantiations of each letter, such that the measured result for each *N*_*c*_ is averaged over 50 × 26 = 1300 experiments. In order to keep the comparison fair, we keep the number of trainable parameters (the total number of output weights of all the ESNs) constant. In practice this means we used 250 neurons for a single controller, 125 for 2 controllers, etc. In Figure 9 we present the results. A single controller performs rather poorly. The optimal number of controllers for the entire English alphabet is around 6 controllers. When the number of controllers increases further, the number of neurons, and hence the modeling power, of each controller becomes smaller. Similar to the experiment with the linear controller, this experiment shows that great deal of the modeling complexity is covered by the mixing mechanism.

It should be noted that in some cases, due to the random initialization, the robot can get stuck in a certain pose (as some joint-angles are limited between certain values), and never reach the desired trajectory. This is the reason why the maximum values in Figure 9 are much bigger than the mean over all its experiments. If we disregard these cases we get an RMSE of 1 mm within 100,000 training samples.

### 3.3. Control primitives

As we have mentioned in the introduction, what we call control primitives in this paper differs from the regular notion of primitives. In this section, we investigate how the actual individual contributions of the controllers behave. Due to the MACOP setup it is not straightforward to get a good understanding of the role of a single controller. At all points in time, all controllers influence the robot, and due to the feedback, each controller influences all other controllers. We can think of two ways in which to study the individual controller contributions. Either we use a single controller in the ensemble (with scaling set to one) for steering the robot, which then ignores potential feedback by the influence of the other controllers, and as such emergent synergies are not expressed. Alternatively, we could record the control signals of the individual controllers during normal operation, and use these recordings to steer the robot afterwards. Even though this approach will take into account potential synergies between the controllers, during testing it has no feedback at all, such that the trajectory could start to drift from the objective. In our setup, however, it seems that such an effect does not occur. Therefore we will use this approach. We have tried the other approach as well, and the results were qualitatively similar.

To get a qualitative idea of how the individual contributions look, we revisit the task inspired on the English alphabet, and train a controller ensemble to draw the letters of the word “amarsi”^4^ one after another. After training, we use the recorded contributions of a single controller (unscaled) to steer the Webots simulation, and record the robot response.

**Figure 10:**
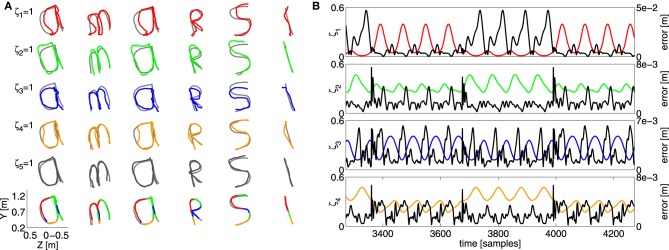
**(A)** Overview of the resulting trajectory with the “amarsi” target by using a single controller contribution. Each row shows the resulting end-effector trajectory of the robot arm. From left to right a part of the continuous writing is shown such that every letter of the word “amarsi” is presented. The coloring of the trajectory illustrates which controllers contribution is used (one for each row). The bottom row show the target trajectory, together with the actual generated trajectory. **(B)** The error of a single controller contribution (the black curves), plotted with their corresponding scaling factors (colored) as a function of time. The vertical scale of the error is provided on the left vertical axis, and that of the scaling on the right.

The result is shown in Figure 10A, the five rows starting from the top show what trajectory each individual control primitive produces if it alone is present in the control architecture (in a corresponding color), plotted over the target value (gray). The bottom row shows the trajectory of the full ensemble, colored according to the most dominant controller. It is interesting to note that, even though all individual controllers produce a trajectory that resembles the target, all of them strongly deviate from the true target, and each of them produces a distinctly different response. The scaled combination of them, however, tracks the objective far more closely, which again indicates that the mixing mechanism works well to combine contributions of several controllers.

A second experiment we perform is to see whether true specialization occurs. After all, even though one controller is dominant, the other controllers will also strongly contribute to the total motion. In order to check this, we have performed a similar experiment to the double-circle objective, depicted in Figure 6. We used four controllers, and used the individual recordings to drive the robot. Next, we record the distance error of the end-effector as a function of time, which we compare with the corresponding scaling factor of the controller. If specialization occurs, we would expect to see some negative correlation between the error on the trajectory and the corresponding scaling of the controller. If the scaling factor of a certain controller is high, this would indicate that it specializes in the current region of task-space, and the resulting error should be low. Vice versa, if the scaling is low the controller should perform worse, as it is not its region of specialization.

The result for each controller is shown in Figure 10B. For some controllers there seems to be a strong relation between the error and the scaling of the corresponding model. Especially in the case of the red and blue controller. The relation is weaker, however, for the other two. Indeed, the scaling factor for these two controllers fluctuate less, such that they are able to train their corresponding inverse models throughout the full trajectory, leading to better overall generalization. From this we can conclude that MACOP uses both specialization and signal mixing to obtain good control over the robot arm.

### 3.4. Coping with dynamic effects

As mentioned before, during training MACOP is capable of handling dynamic effects/transitions caused by the inherent inertia of the robot and the fixed control signal rate. To demonstrate this ability, we apply MACOP to the robot with the square objective we used in Figure 5, but reduce the velocity of each joint. The *P*-parameter in the PID-controllers is reduced from 10 to 2, of which the effect is shown in Figure 4. Furthermore, in order to assess the effect of a sudden jump, we periodically shift the square by half a meter, which introduces discontinuous moments in the objective.

**Figure 11:**
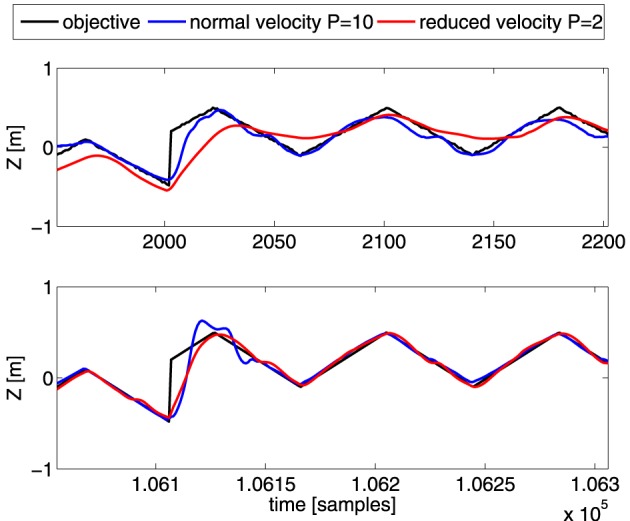
**Comparison of the Z-coordinate of the generated trajectories for the shifted-squares objective.** Shown are the desired Z-coordinate (black), and those generated by robots with *P* = 10 (blue), and *P* = 2 (red). The top panel is during the early parts of the training, and the bottom one after convergence. The abrupt change in the desired trajectory corresponds to the shift of the squares.

In Figure 11 we show the Z-coordinate as a function of time for the desired trajectory and resulting trajectories for the different *P*-parameters. The top panel depicts the results during the beginning of the experiment (30.4–38.4 s), while the bottom panel depicts the results after convergence. The robot with the standard velocity (blue line) is able to follow the objective more closely during the beginning of the experiment but exhibits some fluctuations. The Z-position trajectory of the reduced velocity configuration is unable to reach the objective closely during the first part an clearly needs more time to learn the inverse model, indicating that the control problem is harder when the robot reacts more slowly. If we look at the result after convergence, we notice that the small fluctuations in the blue trajectory are reduced, and that MACOP has learned to follow the objective closely. Interestingly, due to a larger maximum velocity, the blue trajectory has a large overshoot when the applied objective exhibits a sudden jump. The red trajectory exhibits almost no overshoot in the beginning of the experiment but after convergence, the red curve also exhibits some overshoot. In the beginning of the experiment it's clear that the limited velocity of the joints causes the robot's end-effector to not reach its target in time, as opposed to the version with fast control. This indicates that the desired trajectory is more difficult to obtain if the robot has slow dynamics. Indeed, the sudden changes in direction can be made far more easily if the robot has a fast control response, and actuating a single joint may be enough, which is a simple task. If it has a slow response, the robot will need to use synergies between several joints in order to cause the end-effector to switch its direction so suddenly. This makes the control problem far more complicated.

Reducing the velocity of each robot joint has its advantages in terms of power consumption and safety. However, a trade-off must be made between faster convergence, as in closely following the objective, and the amount of overshoot allowed. In some tasks it might be possible that the objective changes very fast, and in such cases, reducing the reaction speed will restrict the robot in reaching its targets.

## 4. Discussion

In this work we described a modular architecture with control primitives (MACOP), which learns to control a robot arm based on a pre-set number of controllers. The inspiration for this architecture stems from MOSAIC (Haruno et al., 2001), which is a control framework, inspired by a plausible model on how human motor control learns and adapts to novel environments. MOSAIC uses a strategy in which an ensemble of inverse-model controllers is trained, one for each environment with different properties. On top of this, a selection mechanism selects which controller needs to be active at which moment in time. Each controller is associated with a forward model of the system that needs to be controlled, and controller selection happens by choosing the forward model which is the most accurate.

Our [and others' (Haruno et al., 2001)] observations show that such a strategy may not be optimal. There is no reason why the accuracy of a forward model should be correlated to that of the inverse model. Another selection mechanism of the controllers might thus be possible. In this work, we want to build upon the idea of a fixed number of control primitives which are continuously combined to produce a desired motion. Each controller used in MACOP consists of an inverse model which is trained online and consists of an Echo State Network. Given some high-level controller mixing requirements, an unsupervised division of the task and joint-space is achieved, which can be related to a Kohonen map (Kohonen, 1998). The mixing mechanism learns a subdivision of the entire task and joint-space and produces one scaling factor for each controller which are associated with the current end-effector and joint-angle position of the robot. The training error of each controller is scaled with the same factor such that training data within a controller's associated part of the subdivision becomes more important than other data. As a result of this data selection mechanism, every controller can specialize its function within its appointed part of the joint and task-space. The used mixing requirements prescribe that all controllers should contribute significantly to the task, while still allowing for a controller to specialize itself for a certain subregion.

We validated MACOP on an inverse kinematic learning task where we controlled a 6 DOF robot arm by producing joint-angles which are sent at a fixed control rate. This is in contrast with other approaches such as Oyama et al. (2001) where a static mapping from task-space position to joint-space is learned and where a separate feedback control loop to approach the target joint-angles is needed. Such a separate feedback control system results in high control gains when their is an external perturbation of the robot's movement. Achieving a compliant kinematic control thus argues for a dynamic learning approach which learns the control at a fixed control rate. In this work we rely on the approach proposed in Waegeman et al. (2012) for such a dynamic control method. As a result MACOP is well suited to cope with the dynamical effects introduced by the non-instantaneous control of the robot: even when the robot responds slowly to the control signal, the MACOP architecture is able to compensate for it and produce the target trajectory.

We replaced each controller with a simple linear controller to validate MACOP's independence of the chosen ESN-controller. Such a linear controller is constructed by learning a linear combination of the architecture's input. When the number of linear controllers within MACOP is large enough, the end-effector will start to track the target. However, the tracking performance of MACOP with the ESN-based controllers is better due to its non-linear nature.

In this work, the contribution of a single controller to all the robot's joint-angles is called a control primitive. Motor and motion primitives generally refer different building blocks at different levels of the motor hierarchy. They can be kinematic (e.g., strokes, sub movements), dynamic (e.g., joint torque synergies, control policies) or both. According to Flash and Hochner (2005) their crucial feature is that a wide variety of movements can be derived from a limited number of stored primitives by applying appropriate transformations. Within this definition a controller's contribution to the joint-angles can be called a primitive. Their organization is stored within the mixing transformation such that after convergence a consistent controller selection is achieved. What is different from the common interpretation of primitives is that in our case, the control primitives are mixed and rescaled constantly, instead of truly being selected and weighted statically.

MACOP learns to spread a set of controllers in the vicinity of the target trajectory such that primitives produced by controllers can help in tracking this trajectory. Even when the complexity of these controller is reduced (using linear controllers), the task is still solvable. Unlike in Nori and Frezza (2004), the MACOP control primitives and their mixing values both depend on the state of the robot, and because they are adapted online they become dependent on the task. After convergence (all learning rates are 0) this task dependency is removed.

In future work we wish to investigate how well MACOP is able to simplify the full dynamic control (e.g., controlling torques) of such systems including real-world robot platforms. Secondly, we wish to investigate how the mixing mechanism adapts to new tasks when the controllers are assumed to be fixed (training completed).

One interesting observation needs more scrutiny. Even though we showed that a single controller is unable to learn the inverse kinematics, we still found that for the controller ensemble, individual controller contributions perform relatively well in tracking the target trajectory. We suspect that this is caused by the used mixing mechanism and its effect on the controller's learning rate. The training data used to update a controller is weighted such that a good local model can be learned more easily instead of polluting the controller with data from other positions. Later, generalization to other positions in task-space can be performed more gradually, simplifying the full training problem. Further tests will be needed to confirm this hypothesis.

### Conflict of interest statement

The authors declare that the research was conducted in the absence of any commercial or financial relationships that could be construed as a potential conflict of interest.

## Footnotes

^1^Here we treat the scaling factors and their target values as if they were probabilities, which stems from the common use of a softmax function: to model a multinomial distribution function (Bishop and Nasrabadi, 2006). We could as well use mean-square error to train **V**(*t*) on the target values, but the resulting update equations would be more complicated, whereas cross-entropy leads to a simple formula.

^2^This is the average distance, such that we can express RMSE in millimeter or centimeter.

^3^This means that the control signal the robot receives can no longer be simply interpreted as joint-angles, but more as a type of motor commands.

^4^AMARSi is an EU project concerning adaptive motor skills for robots.
